# Medical Society

**Published:** 1897-03-27

**Authors:** 


### MEDICAL SOCIETY.

Mr. Harrison Cripps read a paper on Three Cases of Intestinal Anastomosis, two of them involving Double Resection.

In the discussion which followed the interest centred chiefly round two questions—whether in cases of acute obstruction to do immediate resection and anastomosis, or to perform enterotomy, and wait for the urgent symptoms to subside before doing the complete operation; and, secondly, whether to button or to stitch.

Mr. Cripps was very strong on the advantage of the preliminary enterotomy, as not only giving immediate, even if only temporary, relief to a condition which was often so serious as to put out of question any prolonged operation, but as also very much facilitating the operation, when the time for it came, by removing all distension of the bowel above the obstruction, and by enabling the surgeon to take his time, and also as making it much safer, partly because of the absence of inflammatory changes in the peritoneum, and partly because of the fistulous opening made in the preliminary enterotomy, providing a safety valve by which distension by gases of the involved coils of intestine was prevented.

In regard to buttons and bobbins *versus* stitching there is evidently a growing feeling in favour of a surgeon using his own fingers. Mr. Cripps said that he had seen two cases of death from Murphy's button, in one of which, although it did its work properly, it then became wedged, and ulcerated through into the peritoneum, and in the other the whole circle of the bowel embraced by it sloughed off leaving the ends of the bowel open.

Mr. Sheild preferred to trust to his own fingers and a needle, and he urged that surgeons should learn to join bowel to bowel without apparatus. This is a matter of some importance; bowel sewing is an art requiring much deftness of finger, and no inconsiderable practice, and it is an art which must be learned.

